# A novel anti-CD20, concabody, enhances immunotherapy efficacy by targeting MPZL1 and augmenting antibody-induced cell death

**DOI:** 10.3389/fonc.2026.1748576

**Published:** 2026-04-15

**Authors:** A-Yeon Choi, DaHye Kim, Changsin Lee, Jae-Sook Ahn, Deok-Hwan Yang, DongHyuk Lee, Joo Young Kim

**Affiliations:** 1 Department of Pharmacology and Brain Korea 21 Project for Medical Science, Yonsei University College of Medicine, Seoul, Republic of Korea; 2 Department of Hematology-Oncology, Chonnam National University, Chonnam National University Hwasun Hospital, Hwasun, Republic of Korea; 3 Woo Choo Lee Institute for Precision Drug Development, Seoul, Republic of Korea

**Keywords:** APEX2-mediated proximity labeling, B cell lymphoma, concabody, myelinprotein zero like protein 1 (MPZL1), obinutuzumab

## Abstract

**Introduction:**

While anti-CD20 antibodies like obinutuzumab (OBI) have improved clinical outcomes, B-cell malignancies remain a significant therapeutic challenge. OBI induces direct cell death (DCD) and augments antibody-dependent cellular cytotoxicity (ADCC), but the molecular mechanisms involved in DCD remain unclear. This study aims to bridge this knowledge gap and develop enhanced treatment modalities.

**Methods:**

We employed an antibody–APEX2 proximity labeling platform to identify novel regulators of OBI-induced DCD. Mechanistic studies were conducted to evaluate lysosomal membrane permeabilization (LMP). Furthermore, we developed a new class of bioengineered antibody-lectin fusion proteins, termed "concabodies," and evaluated their efficacy through *in vitro* assays and *ex vivo* experiments using peripheral blood mononuclear cells (PBMCs) from patients with chronic lymphocytic leukemia (CLL).

**Results:**

Myelin protein zero-like 1 (MPZL1) was identified as a novel regulator of OBI-induced DCD, modulating LMP as a key characteristic. We found that concanavalin A (Con A) also triggers LMP and DCD via MPZL1-mediated internalization. The developed concabodies exhibited significantly enhanced DCD and ADCC while concurrently reducing the non-specific toxicity of free Con A. Notably, this enhanced cytotoxicity was maintained even in cells expressing a dominant-negative MPZL1^Y241F^ mutant, and the concabodies efficiently depleted malignant B cells in CLL patient samples.

**Discussion:**

Taken together, this work establishes MPZL1 as a crucial molecular target for antibody-induced DCD and presents concabodies as a mechanistically rational and clinically relevant approach to improve the efficacy of anti-CD20 immunotherapy in B-cell malignancies.

## Introduction

Non-Hodgkin lymphoma (NHL) is a prevalent hematological malignancy that accounts for approximately 3% of all global cancer diagnoses and deaths (1, 2). The majority of NHL cases are B-cell non-Hodgkin lymphoma (BNHLs), which arises from clonal expansion of mature or immature B cells (3, 4). According to the World Cancer Research Fund International, BNHL affects over half a million people annually (5, 6).

Anti-CD20 monoclonal antibodies (mAbs) play a central role in BNHL treatment. These antibodies mediate B-cell depletion via multiple mechanisms, including complement-dependent cytotoxicity (CDC), antibody-dependent cellular cytotoxicity (ADCC), antibody-dependent cellular phagocytosis (ADCP), and direct induction of cell death (DCD) induction. Although rituximab (RTX) has significantly improved clinical outcomes, many patients relapse or develop resistance (7–10). In contrast, obinutuzumab (OBI), a humanized, glycoengineered type II anti-CD20 mAb, induces rapid and potent DCD, characterized by homotypic adhesion, actin remodeling, reactive oxygen species (ROS) generation, lysosomal membrane permeabilization (LMP), and lysosome rupture. These processes lead to cathepsin-mediated necrotic cell death, including plasma membrane damage and contribute to superior B-cell clearance compared to RTX, which primarily relies on the CDC (11–14).

To further elucidate the molecular basis of OBI-induced DCD, we employed enzyme-catalyzed proximity labeling (PL) using APEX2, a peroxidase-based system that enables biotinylation of proteins within a ~20 nm radius in live cells (15–20). To achieve this, we developed a recombinant anti-CD20 antibody fused with APEX2, allowing for the specific labeling of proteins in the antigen-proximal region. This method enables the identification of transient or membrane-proximal protein interactions under near-physiological conditions through mass spectrometry. Using our APEX2-based approach and subsequent rational screening, we selected 12 candidate proteins associated with OBI-CD20 complexes and identified myelin protein zero-like 1 (MPZL1), a transmembrane glycoprotein of the immunoglobulin superfamily, as a proximal interactor of CD20 after OBI treatment. MPZL1 is widely expressed and has been implicated in extracellular matrix–induced signaling (21, 22), with oncogenic roles in several cancers (23, 24).

Notably, MPZL1 has been suggested to act as a receptor for concanavalin A (Con A), a plant lectin that binds glycosylated proteins and triggers diverse cellular responses, including autophagy and lysosome-mediated cell death (25–30). However, the functional role of MPZL1 in Con A-induced cytotoxicity remains unclear.

In this study, we aimed to identify the key regulators of OBI-induced DCD and exploit this mechanistic insight to design an improved therapeutic strategy. Guided by the role of MPZL1 in OBI-induced DCD and its interaction with Con A, we engineered a novel fusion protein, the concabody, by linking Con A to anti-CD20 antibodies. The engineered concabodies exhibited enhanced DCD and ADCC while markedly reducing non-specific toxicity. These findings not only clarify the molecular mechanisms of OBI-mediated cell death, but also offer a promising platform for improving the efficacy and selectivity of antibody-based therapies for B-cell malignancies.

## Materials and methods

### Cell lines

All cell lines were routinely tested for mycoplasma contamination and confirmed to be negative throughout the study period. CHO-K1, HEK293T and Ramos cells were purchased from the Korean Cell Line Bank. The Raji cell line was a gift from Prof. Seong Hwan Kim (Chungnam National University, Daejeon). CHO-K1, Ramos and Raji Cells were maintained in Roswell Park Memorial Institute (RPMI) 1640 medium supplemented with 10% fetal bovine serum (FBS) and 1% penicillin/streptomycin (Pen/Strep) at 37 °C and 5% CO_2_. HEK293T cells were maintained in Dulbecco’s modified Eagle (DMEM) high-glucose medium supplemented with 10% FBS and 1% penicillin/streptomycin at 37 °C and 5% CO_2_. For the cell culture, DMEM (Welgene;Cat No.LM001-05), and RPMI 1640 medium (Wellgene; Cat No.LM011-01), (fetal bovine serum FBS (Gibco; Cat No.12483020), penicillin-streptomycin (Wellgene; Cat No. LS202-02), and 0.05% trypsin-EDTA solution (Wellgene; Cat No.LS015-01) was used.

### Lentivirus production

HEK293T cells (8×10^5^ cells/well) were seeded in 6-well plates. After 24 h, 3 µg of target DNA, pMD2G, and psPAX2 were co-incubated with 100 µL of PBS and polyethylenimine (PEI) for 15 min and then added to the cells. The DMEM was changed after 6 h of incubation. The cell culture media were harvested after 48 h, centrifuged at 1000×*g* at 4 °C for 5 min, filtered through a 0.45 µm polyethersulfone (PES) syringe filter, transferred to a clean tube, and stored at -70 °C.

### Recombinant protein production

Sequences of rituximab (MabThera) were obtained from patent publication (US2015O141620A1) (31). The sequence of obinutuzumab (GA101) was obtained from a patent publication (US011110087B2) (32). Anti-CD20 antibody heavy and light chains were cloned into the pLVX recipient vector to produce the lentivirus and transient expression. To make the APEX2-fused antibody expression plasmid, pcDNA3 Connexin43-GFP-APEX2 was used as the template (Addgene; Cat. No.49385). The concanavalin A sequence was synthesized by Bionics(Seoul, Republic of Korea), based on the concanavalin A amino acid sequence reported by Nonis et al. (33).

For anti-CD20 antibody production, we used CHO-K1 cell lines expressing rituximab or obinutuzumab following a previous purification method (34). For APEX2-fused antibody production, 3×10^6^ HEK 293T cells were seeded in a 100 mm culture dish and incubated for 24 h. Ten micrograms of APEX2-fused antibody expression plasmid DNA was co-incubated with 200 µL of PBS and PEI for 15 min and then added to the cells. After 24 h, the culture medium was replaced with Freestyle 293 Expression media (Gibco; Cat No.12338018). The cell culture media were harvested after 120 h and centrifuged at 1000**×** *g* at 4 °C for 5 min. The resulting supernatant was stored at 4 °C until further use. The same purification procedures were used for both the anti-CD20 and APEX2-fused antibodies. Purified protein samples were mixed with 6× Laemmli sample buffer and separated by SDS-PAGE (8-10%) under reducing and non-reducing conditions. Coomassie blue staining was performed for protein quantification and purity validation.

### Proximity labeling using APEX2

The concentration and incubation time of the antibody, biotin phenol, and H2O2 for APEX2 proximity labeling were optimized using 2×10^5^ B cell lymphoma Raji cells. The reactions were conducted in 50 μL of RPMI culture media. For antibody concentration optimization, 7, 21, 35, and 70 nM of each APEX2-fused antibody were treated with 0.5 mM biotin phenol and H2O2. For optimization of incubation time, 35 nM APEX2-fused antibody was added for 15, 30, and 60 min. For biotinylation, 0, 0.25, 0.5, 2.5, and 5 mM of biotin phenol or H2O2 were treated with 0.5 mM of H2O2 or biotin phenol to assess the efficiency of APEX2-dependent biotinylation. Biotinylated proteins were analyzed by western blotting. Based on the optimized APEX2-dependent biotinylation conditions, the samples were prepared for HPLC-MS/MS analysis. Raji cells (1×10^7^) were resuspended in 1 mL RPMI culture medium in conical tubes. Each antibody, including the control antibody (isotype control; obinutuzumab-LC/TRA: trastuzumab [anti-HER2 antibody]-HC), APEX2, RTX:rituximab (anti-CD20 antibody)-APEX2, and OBI:obinutuzumab (anti-CD20 antibody)-APEX2, was added to a separate tube and incubated at 37 °C for 30 min. Meanwhile, 2× and 1× quenching solutions (0.5 mM MgCl_2_, 1 mM CaCl_2_, 5 mM Trolox, 10 mM sodium azide, 10 mM sodium ascorbate) and a 9.8 mM H_2_O_2_ solution in PBS were prepared. The cells were centrifuged at 1000**×** *g* for 5 min and the supernatant was removed. The cells were washed twice and resuspended in 1 mL pre-warmed RPMI culture media containing 0.5 mM biotin phenol. After 1 minute, a 0.5 mM H_2_O_2_ solution was added to each tube, followed by incubation for 1 min. To quench the biotinylation reaction, 1 mL of the 2× quenching solution was added to each tube. Biotinylated samples were centrifuged at 1000× *g* for 5 min, the supernatant was removed, and the cells were gently mixed with 1 mL of 1× quenching solution. After another centrifugation, the supernatant was discarded and the cells were resuspended in 1 mL of RIPA buffer containing quenching reagents. The mixture was then incubated at 4 °C for 20 min. The protein lysates were centrifuged at 18000×*g* at 4 °C for 10 min, and the supernatants containing biotinylated proteins were carefully transferred to new tubes. Streptavidin agarose beads were used to enrich the biotinylated proteins. The supernatant containing biotinylated proteins was incubated with beads at 4 °C with end-to-end rotation. After 2 h, the tubes were centrifuged at 500**×** *g* for 2 min and washed with 1 mL of washing buffer (1 M KCl, 0.1 M Na_2_CO_3_, 2 M urea in 10 mM Tris-HCl pH 8 in 1 mL of RIPA buffer). The beads were then washed repeatedly with 1 mL of RIPA buffer without detergents (10 mM Tris-HCl pH 7.5, 150 mM NaCl, 0.5 mM EDTA). On-bead digestion was performed to elute biotinylated proteins. The beads were incubated with 25 μL of elution buffer 1 (50 mM Tris-HCl pH 7.5, 2 M urea, 5 μg/mL trypsin, and 1 mM DTT) in a thermomixer at 30 °C with shaking at 400 RPM shaking. After 30 min, the beads were centrifuged at 2500**×** g for 2 min at 4 °C, the supernatant was transferred to a new tube, and 25 μL of elution buffer 2 (50 mM Tris-HCl pH 7.5, 2 M urea, 5 mM iodoacetamide) was added to the beads, which were then centrifuged at 2500× g at 4 °C for 2 min twice. The eluates from both steps were combined and incubated in a thermomixer at 32 °C with shaking at 400 RPM shaking overnight to continue digestion (protected from light). After 12 h, the reaction was quenched by adding 1 μL trifluoroacetic acid (TFA).

### High-performance liquid chromatography – mass spectrometer analysis

Trypsinized samples from streptavidin agarose beads were used for HPLC-MS/MS analysis using a Bruker TimsTOF Pro 2 mass spectrometer (Bruker Daltonics, Bremen, Germany). Peptide identification and intensity analysis were performed using the PEAKS software (Bioinformatics Solutions Inc.). The HPLC-MS/MS analysis was conducted by the Korea Basic Science Institute (KBSI, Seoul, South Korea). For qualitative analysis, the final protein target lists were filtered from the analyzed data using the following strategies:

1. The intensity of each detected protein was normalized to the total intensity acquired during the analysis. The lists were ordered by intensity in descending order.
2. The normalized intensity from OBI-APEX2 (O-A) and RTX-APEX2 (R-A) was divided to calculate the fold change between the two anti-CD20 antibodies.
3. Proteins detected by more than two unique peptides were selected.
4. Proteins overlapping with control-APEX2 (C-A) were discarded.
5. Proteins showing a greater than 1.4-fold change were selected.
6. Finally, plasma membrane proteins were selected as the final protein targets.

Candidate gene information was acquired by Genecards.org (35, 36), and protein information was acquired by UniProt.org (37). Gene ontology and classification were analyzed using Metascape.org (38).

### Western blotting

Protein samples were obtained from an equal number of cells in RIPA buffer. The samples were mixed with 6× Laemmli sample buffer and 20 μL of each sample was loaded onto an acrylamide gel for protein separation by electrophoresis. The separated proteins were transferred onto Polyvinylidene Fluoride (PVDF) membranes. The membrane was activated with methanol and blocked with TBS-T containing 5% non-fat milk and 0.1% Tween 20 for 1 h at room temperature. The membranes were then incubated with the primary antibody for 1 h at room temperature. After washing the membrane thrice with TBS-T for 10 min each, the secondary antibody was added and incubated for 1 h at room temperature. Following three additional washes with TBS-T for 10 min each, proteins were detected using ECL solution (Cytiva; Cat No.RPN2232).

### Silver staining

The lysates obtained from the same number of cells in RIPA buffer were mixed with 6× lammeli sample buffer, and 20 μL of each sample was separated on pre-cast 10% SDS PAGE gels in both reducing and non-reducing conditions. The gel was stained using the Pierce TM Silver Stain Kit, according to the manufacturer’s protocol (ThermoFisher Scientific; Cat No.24612).

### Cell binding assay

Raji cells (2**×**10^5^) were resuspended in 50 μL of PBS. The cells were then incubated with the indicated concentrations of anti-CD20 antibodies (OBI, RTX), anti-CD20-APEX2 fusion antibodies (O-A, R-A), and control antibodies (C-A) at 4 °C for 30 min. After washing the cells with PBS, the cell pellets were resuspended in 50 μL of PBS containing an anti-human Ig Fc-specific FITC-conjugated secondary antibody at a 1:500 dilution (Jackson Laboratories; Cat No.109-095-008). The cells were incubated at 4 °C for 30 min. After washing the cells twice with PBS, 10,000 cells were counted by flow cytometry (FACS LSR II SORP system, BD Biosciences) and analyzed using FlowJo™ v10.8 Software (BD Life Sciences). For the concanavalin A-binding assay, cells were incubated with 1 μg/mL biotinylated concanavalin A (Sigma-Aldrich; Cat No.C2272) for 30 min. Anti-streptavidin APC (allophycocyanin) was added at 1:500 dilution and incubated for 30 min.

### Measurement of lysosomal membrane permeabilization

Raji or Ramos cells (1**×**10^5^ cells/well) were stained with LysoTracker Deep Red (Invitrogen; Cat No.L12492) for 30 min at 37 °C. For the Con A (Sigma-Aldrich; Cat No.C5275) LMP assay, the cells were treated with Con A at the indicated concentrations. After incubation, the cells were stained with LysoTracker Green (Invitrogen, Cat No.L7526) at 37 °C for 30 min. To quantify LMP, we established a fluorescence gate based on the baseline signal of the untreated control cells, which represented the intact lysosomal population. The degree of LMP was then determined by calculating the percentage of cells that shifted out of this pre-defined normal range following treatment with varying concentrations of ConA. This approach allowed for a precise assessment of the sub-population undergoing lysosomal destabilization in response to concabody treatment. The geometric mean (Geomean) of LysoTracker fluorescence was calculated by flow cytometry(FACS LSR II SORP system, BD Biosciences)and analyzed using FlowJo™ v10.8 Software (BD Life Sciences).

### Direct cell death assay

Raji or Ramos cells (1×10^5^ cells/well) were resuspended in 100 μL of RPMI full medium and the indicated doses of antibody (7, 21, 35, and 70 nM) for 6 h. For the Con A DCD assay, cells were treated with Con A at the indicated concentrations. Then, the cells were stained with 1 μL of 1 mg/ml propidium iodide (PI) or 0.5 μL of 0.25 μg/mL Calcein-AM at 37 °C for 30 min. The PE-positive or FITC-negative cells were used as a readout of cell death (% of fluorescence exposing cell number among 10,000 counted total cells), which was detected by flow cytometry(FACS LSR II SORP system, BD Biosciences)and analyzed using FlowJo™ v10.8 Software (BD Life Sciences).

### Internalization assay

Raji cells (2×10^5^ cells/well) were resuspended in 50 μL of PBS containing the indicated concentrations of Con A, antibodies, or concabodies, and incubated on ice at 4 °C for 30 min. After incubation, the cells were washed once with cold PBS to remove unbound components and subsequently incubated with 10,000 MW ((Sigma-Aldrich; Cat No.FD10S) at a final concentration of 100 μg/mL at 37 °C for 4 h in a CO_2_ incubator. Following endocytosis, cells were washed with cold PBS to remove extracellular dextran. The fluorescence intensity of internalized FITC-dextran was detected by flow cytometry(FACSymphony A5, BD Biosciences), and 10,000 events per sample were acquired and analyzed using FlowJo™ v10.8 Software (BD Life Sciences).

### Doxycycline induced-knockdown and CRISPRa-mediated overexpression

A doxycycline-induced shRNA-dependent knockdown system was developed by cloning lentiviral vectors containing the target shRNA antisense guide sequences. Antisense guide RNA sequences targeting the UTR were obtained from the SplashRNA website (http://splashrna.mskcc.org/) and amplified using PCR (**Table 1**). The amplified products were then cloned into the LT3GEPIR plasmid with a pRRL backbone (Addgene; Cat No.111177) using XhoI/EcoRI sites. Lentiviruses were produced as described earlier, transduced into 5×10^4^ Raji cells, and selected with 2 μg/mL of puromycin. After selection, 1 μg/mL doxycycline was added to 1×10^6^ shRNA-induced Raji cells, and GFP-positive cells were isolated using a sorter (FACS Aria III, BD Biosciences). To validate doxycycline-induced target gene silencing, 3**×**10^6^ shRNA-induced Raji cells were treated with 0.1 μg/mL doxycycline at a concentration of 3 days. RNA was isolated by adding 500 μL of TRIzol reagent to the cells, incubating the mixture at room temperature for 5 min, and adding 100 μL of chloroform. The mixture was vortexed vigorously and incubated at room temperature for 3 minutes. The samples were centrifuged at 13,000× *g* at 4 °C for 15 min. The colorless upper aqueous phase was carefully transferred to a new tube and incubated with 250 μL of isopropanol at room temperature for 10 min. The solution was centrifuged at 13,000**×** *g* and 4 °C for 10 min, and the RNA pellet was washed twice with 500 μL of cold 75% ethanol. The RNA pellet was centrifuged at 7500**×** *g* for 5 min and dried under vacuum for 5 min to remove any residual ethanol. The RNA pellet was dissolved in 50 μL DEPC-treated water and quantified using a NanoDrop™ Lite Spectrophotometer (Thermo-fisher). The extracted RNA was reverse-transcribed using the SuperiorScript III cDNA Synthesis Kit (Enzynomics; Cat No.EZ405S). The obtained cDNA was used to quantify knockdown efficiency using a QuantStudio 3 Real-Time PCR instrument (Applied Biosystems) with AccuPower^®^ 2×GreenStar qPCR master mix (Bioneer; Cat No.K-6251). The PCR conditions were as follows: 95 °C for 2 min (initial denaturation), followed by 40 cycles of 95 °C for 10 s and 58 °C for 30 s (denaturation, annealing, elongation, and measurement). A melt-curve step was performed according to the default settings of the machine manual. Data analysis was performed by calculating the ΔΔCT values between GAPDH and the target genes (39).

**Table 1:** Sequence of shRNAs.

Target gene	shRNA oligo sequence
1. *F11R*	TGCTGTTGACAGTGAGCGCGCATAGTAAATTTTCAGAGAATAGTGAA GCCACAGATGTATTCTCTGAAAATTTACTATGCTTGCCTACTGCCTCGGA
2. *CD40*	TGCTGTTGACAGTGAGCGAAGCATTGTTTGTGATAGTGAATAGTGAA GCCACAGATGTATTCACTATCACAAACAATGCTGTGCCTACTGCCTCGGA
3. *MPZL1*	TGCTGTTGACAGTGAGCGCGTGTACAAAGGATATGTATAATAGTGAA GCCACAGATGTATTATACATATCCTTTGTACACATGCCTACTGCCTCGGA
4. *CD82*	TGCTGTTGACAGTGAGCGCGCCTGTATCAAAGTCACCAAATAGTGAA GCCACAGATGTATTTGGTGACTTTGATACAGGCTTGCCTACTGCCTCGGA
5. *ST14*	TGCTGTTGACAGTGAGCGCCCAACTCCACTGAGTTTGTAATAGTGAA GCCACAGATGTATTACAAACTCAGTGGAGTTGGATGCCTACTGCCTCGGA
6. *SLAMF6*	TGCTGTTGACAGTGAGCGACAGAGTAATGTTTTTAATATATAGTGAA GCCACAGATGTATATATTAAAAACATTACTCTGGTGCCTACTGCCTCGGA
7. *TSPAN14*	TGCTGTTGACAGTGAGCGCAGTGTGTAAATAGTTTATTTATAGTGAA GCCACAGATGTATAAATAAACTATTTACACACTTTGCCTACTGCCTCGGA
8. *BSG*	TGCTGTTGACAGTGAGCGACCAGTGCTTGCAAGATTCCAATAGTGAA GCCACAGATGTATTGGAATCTTGCAAGCACTGGGTGCCTACTGCCTCGGA
9. *TRIM21*	TGCTGTTGACAGTGAGCGCCCACTGAATATTGGATCACAATAGTGAA GCCACAGATGTATTGTGATCCAATATTCAGTGGATGCCTACTGCCTCGGA
10. *HLAE*	TGCTGTTGACAGTGAGCGATCCGAGCAAAAGTCAAATGATTAGTGAA GCCACAGATGTAATCATTTGACTTTTGCTCGGAGTGCCTACTGCCTCGGA
11. *CORO1A*	TGCTGTTGACAGTGAGCGCCAGTGGCTTCTGTGCTGTCAATAGTGAA GCCACAGATGTATTGACAGCACAGAAGCCACTGTTGCCTACTGCCTCGGA
12. *RAB11A*	TGCTGTTGACAGTGAGCGACAGGTCTGAGATTTAAATATATAGTGAA GCCACAGATGTATATATTTAAATCTCAGACCTGGTGCCTACTGCCTCGGA

Sequence of shRNAs.

For overexpression of MPZL1 using CRISPRa, lentiMPH v2 (Addgene Cat No.89308) and lentiSAMv2 (Addgene; Cat No.75112) were used, and sgRNA sequences for the guide RNA were designed using CRISPick (Broad Institute, https://portals.broadinstitute.org/gppx/crispick/public). Expression was confirmed by qPCR and western blotting.

### Antibody dependent cellular cytotoxicity assay

ADCC assays using human PBMCs as effector cells were performed as previously described (11, 40), with minor modifications to optimize the effector-to-target (E:T) ratios and incubation times. Peripheral blood mononuclear cells (PBMCs) in 6 mL of blood with 6 mL of PBS were layered onto 6 mL of Ficoll-Paque PLUS (Sigma-Aldrich; Cat No.Histopaque-1077) and centrifuged at 400**×** *g* at 20 °C for 30 min to isolate white blood cells. The white blood cell layer was collected and washed three times with RPMI-1640 medium to remove platelets. Raji cells were washed with PBS and stained with 0.25 μM calcein-AM. The cells were incubated for 30 min and washed twice with the RPMI medium. Purified PBMCs (E:T ratio of 4:1) were added to Raji cells and the cells were treated with the indicated doses of antibodies and incubated at 37 °C in 5% CO_2_ for 4 h. The percentage of FITC^+^ cells among 60,000 PBMCs was determined by flow cytometry (FACSymphony A5, BD Biosciences) and analyzed using FlowJo™ v10.8 Software (BD Life Sciences).

ADCC activity was normalized as follows: ADCC % = 100*[(untreated FITC-positive cell population-treated FITC-positive cell population)/untreated FITC-positive cell population].

### Chronic lymphocytic leukemia patient B cell depletion assay

Samples from three CLL patients obtained from the Biobank of Hwasun Chonnam National University Hospital. CLL patient cells (1×10^5^ cells/well) were prepared and resuspended in 100 μL of RPMI full media with 2.1 nM of antibodies for 6 h at 37 °C in a CO_2_ incubator. B cells were stained with an allophycocyanin (APC)-anti-human CD19 antibody (BioLegend; Cat No.392504). The percentage of B-cell depletion was determined by flow cytometry (FACSymphony A5, BD Biosciences) and analyzed using FlowJo™ v10.8 Software (BD Life Sciences). Normalization was performed as follows: B cell depletion = 100*[(untreated APC-positive cell population-treated APC-positive cell population)/untreated APC-positive cell population]. Experiments were performed using samples derived from three different patients (n = 3), with each sample analyzed in technical triplicate.

### Expression of MPZL1^Y241F^ dominant negative mutant

The MPZL1 clone (hMU008848) was provided by the Korea Human Gene Bank Medical Genomics Research Center, KRIBB, Korea. The MPZL1Y241F dominant mutation was generated by PCR amplification and the PCR product was ligated into the pLVX vector to generate the lentivirus. To produce lentiviral particles, HEK293T producer cells were co-transfected with the MPZL1^Y241F^ expression vector and the packaging plasmids psPAX2 and pMD2.G. The viral supernatants were collected, filtered, and subsequently used to transduce target Raji cells (5×10^4^). Following transduction, stable cell lines were established through selection with 2 μg/mL puromycin.

### Confocal microscopy and colocalization analysis

For colocalization analysis, Raji cells (5x10^5^ cells) were incubated with the indicated antibodies (70 nM) in 100 μL of complete culture medium at 37 °C for 30 min. After incubation, cells were fixed with 4% paraformaldehyde, permeabilized with 0.2% Triton X-100, and blocked with 2% BSA in PBS. To visualize the localization of the CD20 antibodies, cells were stained with Alexa Fluor^®^ 647-conjugated AffiniPure^®^ Goat Anti-Human IgG (H+L) (Jackson ImmunoResearch; Cat. No.109-605-003). For MPZL1 detection, cells were incubated with PZR (D17B10) Rabbit mAb (Cell Signaling Technology; Cat No.9893), followed by Alexa Fluor™ 488-conjugated Goat anti-Rabbit IgG (H+L) Cross-Adsorbed Secondary Antibody (Thermo Fisher Scientific; Cat No.A-11008). Fluorescence images were captured using an LSM 700 confocal microscope (Carl Zeiss, Oberkochen, Germany). Colocalization analysis was performed using the Coloc2 plugin in Fiji(ImageJ) software (41).

### Statistical analysis

Statistical analyses were performed using Prism v9 (GraphPad Software). Data are presented as means ± standard deviation from n independent biological replicates. Technical replicates, when used, are indicated separately. Statistical significance was assessed using Welch’s multiple unpaired t-tests. For comparisons between multiple conjugate groups, One-way ANOVA followed by Tukey’s *post-hoc* test was employed. Statistical significance for Welch’s t-test is indicated by asterisks (*), while significance for ANOVA is denoted by hash marks (#).

Significance is indicated as follows: *p < 0.05, **p < 0.01, ***p < 0.001, ****p < 0.0001. #p < 0.05, ##p < 0.01, ###p < 0.001, ####p < 0.0001.

## Results

### Developing APEX2-fusion antibodies and optimizing proximity labeling

**Figure 1:**
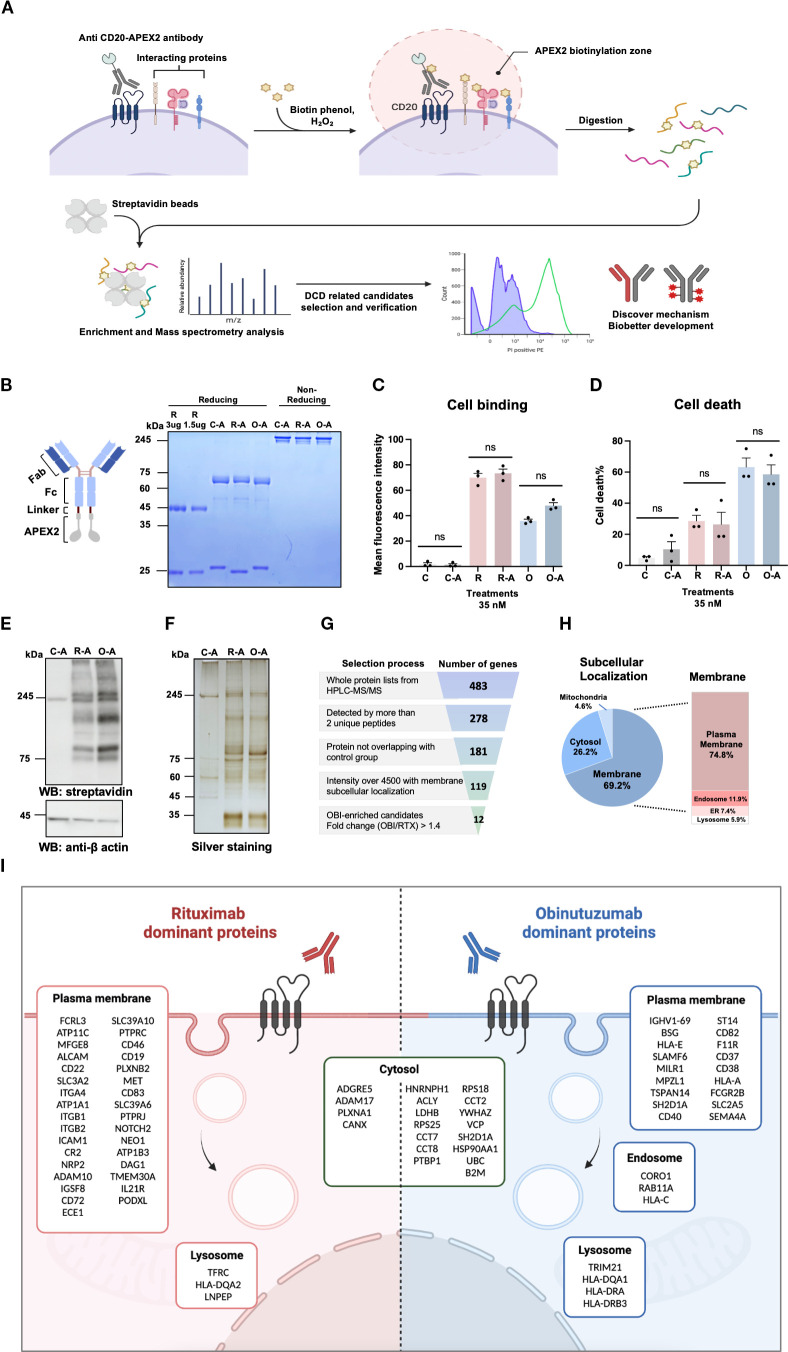
Workflow of APEX2-mediated labeling to identify proteins near obinutuzumab-bound CD20. **(A)** Schematic overview of the APEX2-based proximity-labeling system and experimental workflow to identify proteins in the vicinity of the anti-CD20 mAb–CD20 complex. **(B)** Design of APEX2-fused recombinant antibodies and validation of the purified antibodies by SDS-PAGE. These recombinant APEX2-fused antibodies carry a 4×G4S linker (~2kDa) between the Fc region and APEX2 (2nd generation APEX2 design). R, rituximab; C-A, isotype control (OBI-LC/TRA-HC)-APEX2; R-A, rituximab-APEX2; O-A, obinutuzumab-APEX2. **(C)** CD20 binding ability of wild-type and APEX2-fused anti-CD20 mAbs in Raji cells (n=3). **(D)** Antibody-induced direct cell death (DCD) activity of the wild-type and APEX2-fused antibodies in Raji cells (n=3). **(E)** Western blot analysis of biotinylated proteins using APEX2-fused antibodies was performed in Raji cells. Representative blots are shown (n=2). **(F)** Silver staining of biotinylated proteins. Representative blots are shown (n=2). **(G)** Pipeline for candidate protein selection from proximity-labeling mass spectrometry data. **(H)** Subcellular localization of identified candidate Proteins. Localization was annotated using the GeneCards and NCBI databases. **(I)** Subcellular localization of proteins predominantly enriched by rituximab and obinutuzumab. All data are presented as the mean ± SD. Statistical analysis was performed using the Welch’s t-test. Significance is indicated as follows: ns, not significant.

To identify proteins associated with CD20 upon antibody binding to the cell surface, we developed anti-CD20-APEX2-mediated proximity labeling in combination with mass spectrometry (**Figure 1A**). Initially, we directly fused APEX2 to the C-terminus of the heavy chain Fc region of obinutuzumab (OBI-APEX2) and rituximab (RTX-APEX2). An OBI light chain (LC) N97A mutation was generated as a negative control antibody (Obi^m^-APEX2) (42) (**Supplementary Figures S1A, B**). Despite retaining the ability to bind CD20, the first generation of fusion proteins exhibited a reduced affinity compared to wild-type antibodies (**Supplementary Figure S1B**). We addressed this by introducing a 4×G4S linker between the Fc region and APEX2 which rescued the fusion proteins’ biological functions, such as cell binding and DCD induction, to a level similar to wild-type antibodies. This second generation of linker-modified fusion proteins were designated C-A (isotype control), R-A (rituximab), and O-A (obinutuzumab) (**Supplementary Figure S1C**; **Figures 1B–D**). Since the negative control (Obi^m^-APEX2) unexpectedly showed remaining binding affinity to CD20, we also generated another isotype control by combining the OBI-light chain and trastuzumab-heavy chain (C-A). As expected, the control chimera showed no specific binding to CD20 in Raji cells (**Figure 1C**). We purified the second-generation APEX2-fused recombinant antibodies and used them for further experiments. (**Figures 1B–D**).

Next, to optimize proximity labeling efficacy and plasma membrane integrity, we fine-tuned conditions such as biotin-phenol and hydrogen peroxide (H_2_O_2_) concentrations as well as antibody incubation time. The optimal conditions were determined to be 0.5 mM biotin-phenol, 0.5 mM hydrogen peroxide, 35 nM antibody, and 30 min of incubation (**Supplementary Figure S2**). As shown in **Figures 1E, F**, APEX2-mediated biotinylation and patterns of eluted proteins for each antibody were analyzed by western blotting and silver staining, respectively, under these conditions. Subsequently, on-bead digestion of the enriched samples was conducted for further HPLC-MS/MS analysis, and the protein lists from each antibody are summarized in **Supplementary Table S1**. To identify the potential candidates involved in DCD, we employed a multistep selection process. We normalized peptide intensities, calculated fold changes between O-A and R-A treated samples and selected proteins that detected at least two unique peptides. Additionally, we prioritized plasma membrane proteins and fold changes greater than 1.4. This strategy yielded a subset of 12 candidate proteins for further investigation (**Figure 1G**). Rituximab and obinutuzumab-specific proteins were primarily localized to the plasma membrane, which is consistent with our experimental design (**Figures 1H, I**).

### Validation of candidate proteins in OBI-induced lysosomal membrane permeabilization and selection of novel therapeutic target, MPZL1, for enhancing antibody-mediated DCD

**Figure 2:**
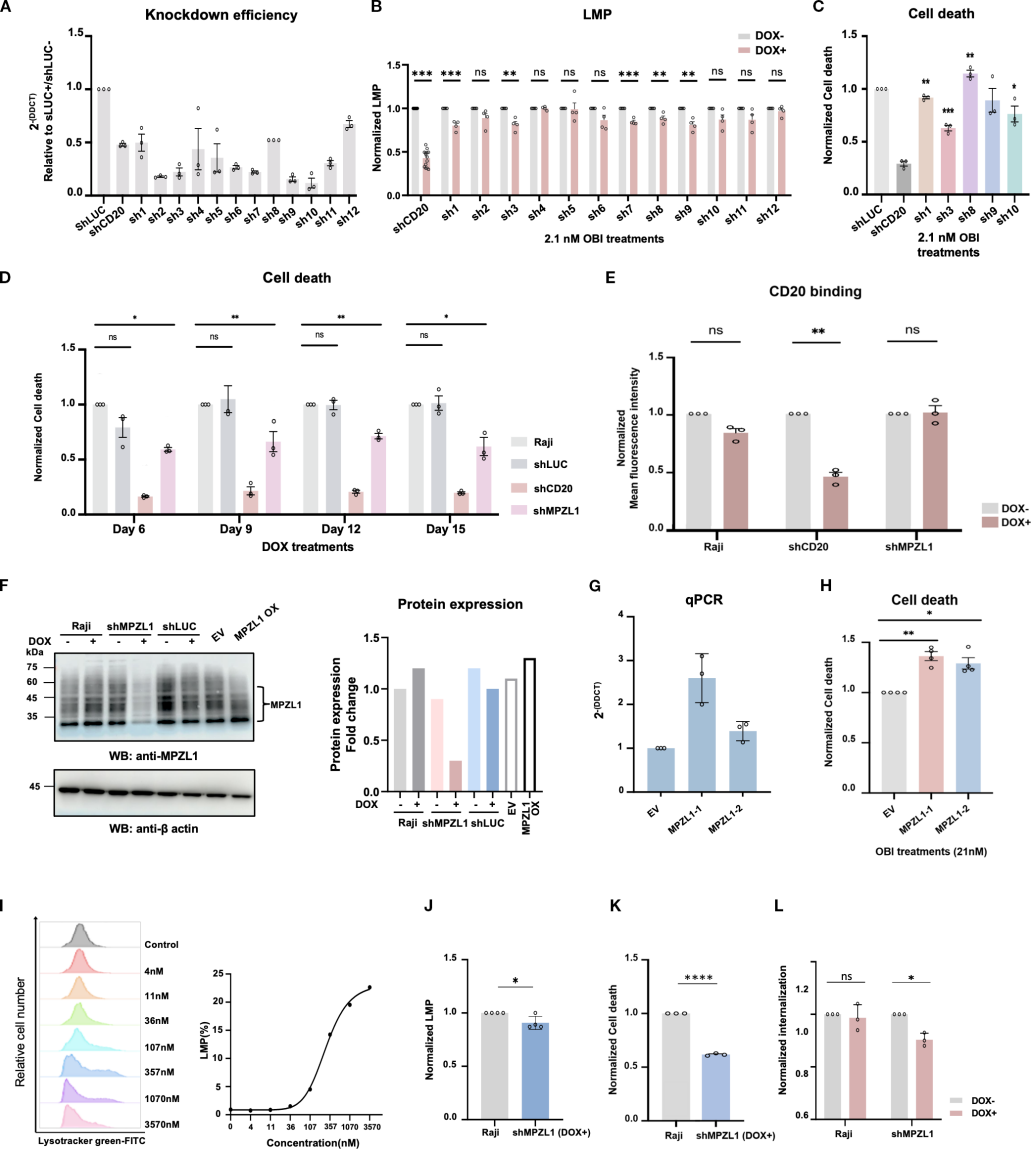
MPZL1 is a novel therapeutic target identified through its role in OBI-induced lysosomal membrane permeabilization and direct cell death. **(A)** Validation of doxycycline-inducible gene knockdown in Raji cells using qPCR (n=3). shRNAs targeting the indicated genes were induced with doxycycline; shLUC (luciferase) and shCD20 were used as negative and positive controls, respectively. **(B)** Quantification of lysosomal membrane permeabilization (LMP) following OBI treatment in candidate knockdown cells (n=4). LMP was measured as the proportion of cells exhibiting reduced LysoTracker Green fluorescence. DOX+, doxycycline-induced knockdown; DOX−, control. **(C)** Measurement of OBI-induced direct cell death (DCD) in candidate knockdown cells showing altered LMP, compared to shLUC controls (n=3). DCD was assessed by propidium iodide (PI) staining. **(D)** Time-course analysis of OBI-induced DCD (21 nM) in MPZL1 knockdown cells (sh3 from panel C) following doxycycline induction(n=3). **(E)** Analysis of CD20 expression by RTX binding in the MPZL1 knockdown cell line(n=3). **(F)** Western blot validation of MPZL1 knockdown (DOX+) and overexpression (MZPL1 OX) by CRISPRa. EV, empty vector. Band intensities were quantified using ImageJ. Representative blots are shown (n=2). **(G)** qPCR analysis of MPZL1 expression in MPZL1-overexpressing cell lines (n=3). **(H)** Enhanced OBI-induced DCD in MPZL1-overexpressing cell lines (n=3). **(I)** Dose-dependent LMP induced by concanavalin A (Con A) in Raji cells. **(J–L)** Reduced Con A-induced LMP **(J)**, DCD **(K)**, and Con A-internalization **(L)** in MPZL1 knockdown cell lines (n=3). Con A (100nM) was used in all three panels. Data are presented as mean ± SD. Statistical analysis was performed using Welch’s t-test. Significance is indicated as follows: ns, not significant; *p < 0.05; **p < 0.01; ***p < 0.001; ****p < 0.0001.

To evaluate the role of the 12 selected protein candidates in OBI-induced DCD, we used doxycycline (DOX)-inducible shRNA to create knockdown (KD) Raji cell lines (sh1 to sh12). In these cells, we measured both LMP, a key characteristic of OBI-induced DCD, and cell death. DOX-treatment for 6 days induced shRNA, and the lowered expression levels of target genes were quantified by real-time PCR (**Figure 2A**). Including the CD20 KD cell line (shCD20) as a positive control, five candidates showed significant differences in LMP levels between the DOX-untreated and treated cells (DOX-/+) after OBI treatment (**Figure 2B**). To confirm these proteins’ role in DCD and to assess their potential as novel therapeutic targets, we performed the DCD assay. As a result, the MPZL1 knockdown (sh3) showed the most significant reduction in cell death among all the tested knockdown lines, a finding consistent with the LMP results(**Figure 2C**). The reduced DCD levels were maintained when doxycycline was administered for longer days (**Figure 2D**). Since CD20 binding showed no difference between normal and MPZL1 knockdown cells (**Figure 2E**), the observed changes in LMP and DCD levels were attributed to MPZL1 expression and its functions rather than antibody binding itself. In contrast, mirroring the reduced DCD observed upon MPZL1 knockdown, overexpression of MPZL1 using CRISPRa enhanced DCD, as anticipated. The overexpression of MPZL1 was validated by real-time PCR and western blotting (**Figures 2F–H**). Thus, based on its significant effect on OBI-induced DCD, we selected MPZL1 as a new therapeutic target for further studies to enhance the function of DCD in anti-CD20 antibodies. To further validate the APEX2 screening results and investigate the spatial relationship between MPZL1 and anti-CD20 antibodies, we performed immunofluorescence (IF) microscopy (**Supplementary Figure S3**). Our observations confirmed that MPZL1 colocalizes with anti-CD20 antibodies at the plasma membrane. Notably, OBI-induced colocalization was significantly enhanced compared to that of RIT, a finding that is highly consistent with our APEX2-mediated proximity labeling data. Taken together, these results provide robust evidence that MPZL1 selectively interacts with the OBI-CD20 complex at the cell surface, potentially facilitating the subsequent DCD signaling pathway.

Previous reports have demonstrated that MPZL1 is a surface glycoprotein involved in signal transduction and tumor progression and is one of the receptors for the lectin concanavalin A (Con A) (25–27). ConA is known to induce autophagic and lysosomal rupture-dependent cell death through internalization (29, 30, 43). However, the role of MPZL1 in Con A-mediated lysosomal rupture and the broader biological role of Con A in B-cell lymphoma have not yet been investigated. To investigate this, we first tested whether Con A induces LMP in Raji cells in a dose-dependent manner. As shown in **Figure 2I**, Con A treatment caused a dose-dependent increase in LMP, confirming its ability to induce lysosomal damage, similar to OBI. Interestingly, we found that Con A internalization, as well as Con A-induced LMP and cell death, were reduced in MPZL1-knockdown cells (**Figures 2J–L**). These findings suggest that MPZL1 plays a critical role in promoting Con A internalization, LMP, and cell death.

### Development and functional characterization of concabody

Based on our findings, we hypothesized that fusing Con A with anti-CD20 antibodies could enhance antibody-mediated cell death while minimizing the non-specific toxicity typically associated with Con A. Given the broad glycan-binding capacity of Con A, we reasoned that coupling it to a high-affinity antibody would allow for targeted delivery to CD20-expressing cells, thereby reducing off-target exposure and limiting non-specific cytotoxic effects.

**Figure 3:**
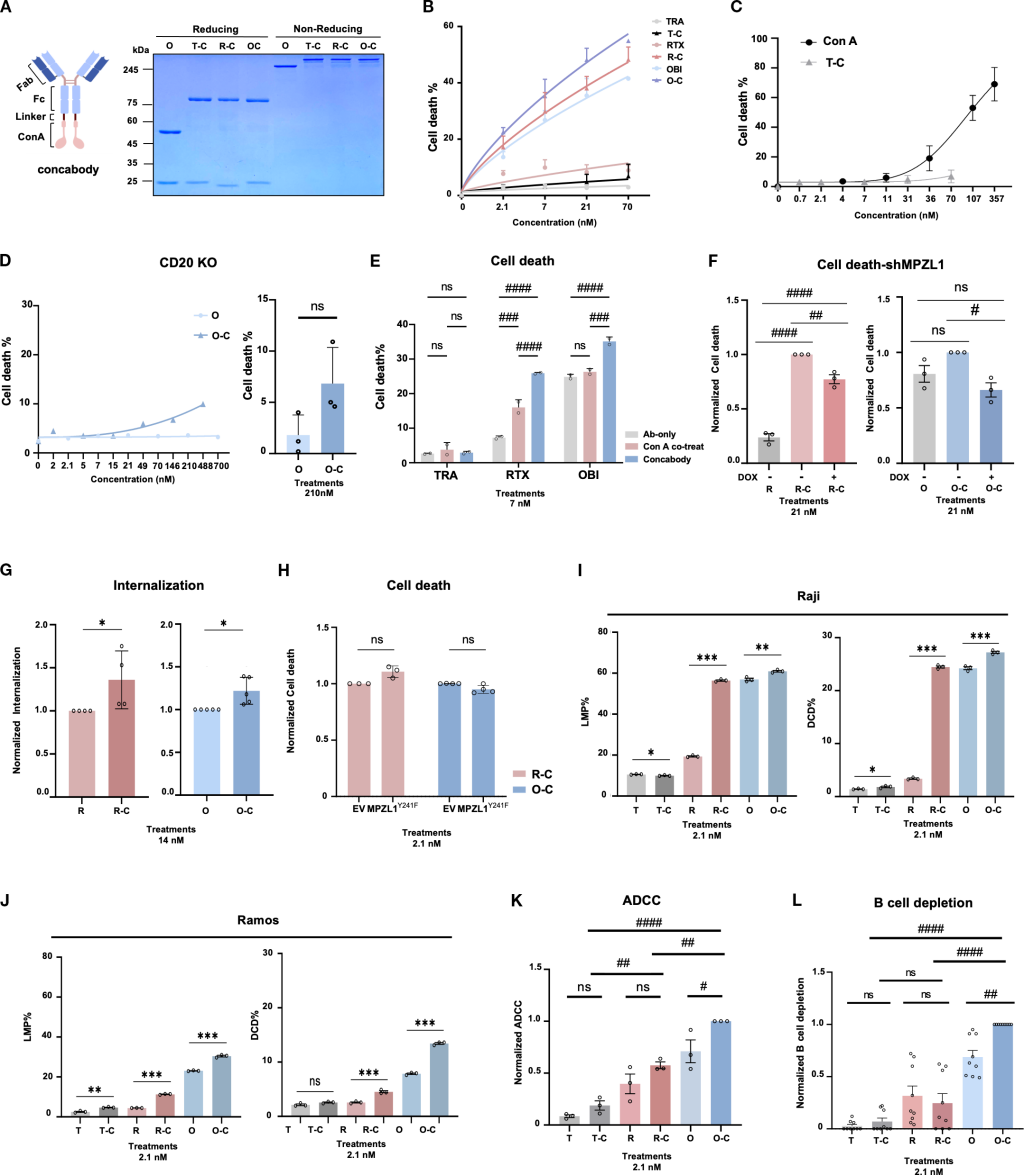
Concabodies, anti-CD20 antibodies fused with the MPZL1-binding lectin Con A, enhance direct cell death and antibody-dependent cellular cytotoxicity. **(A)** Schematic design of concabody and SDS-PAGE analysis of purified concabodies. These recombinant Con A–fused antibodies carry a 4×G4S linker(~2kDa) between the Fc region and Con A (~28 kDa). O, obinutuzumab; T-C, isotype control (trastuzumab)-Con A; R-C, rituximab-Con A; O-C, obinutuzumab-Con A. **(B)** Dose-dependent induction of direct cell death (DCD) by wild-type antibodies (TRA, RTX, OBI) and concabodies (T-C, R-C, O-C) in Raji cells (n=3). **(C)** Cell death induced by TRA-Con A or free Con A in Raji cells(n=3). **(D)** DCD induced by OBI and OBI-Con A in CD20 knockout Raji cells. **(E)** Comparison of DCD induced by wild-type anti-CD20 antibodies alone, free Con A plus antibody co-treatment, and concabodies in Raji cells (n=3). **(F)** MPZL1 dependency of R-C– and O-C–induced DCD assessed in doxycycline-inducible MPZL1 knockdown cells (DOX− *vs*. DOX+) in Raji cells(n=3). **(G)** Internalization efficiency of concabodies compared to wild-type antibodies in Raji cells (n=4). **(H)** DCD induced by concabodies in MPZL1^Y241F^ mutant cells, in which phosphorylation-dependent signaling is disrupted in Raji cells (n=3). EV, empty vector. **(I)** Lysosomal membrane permeabilization(LMP) (left) and direct cell death(DCD) (right) assays were conducted in Raji cells using wild-type antibodies (T, TRA; R, RTX; O, OBI) and concabodies (T-C, TRA-Con A; R-C, RTX-Con A; O-C, OBI-Con A)(n=3). **(J)** Lysosomal membrane permeabilization(LMP) (left) and Direct cell death(DCD) (right) assays were conducted in Ramos cells using wild-type antibodies (T, TRA; R, RTX; O, OBI) and concabodies (T-C, TRA-Con A; R-C, RTX-Con A; O-C, OBI-Con A)(n=3). **(K)** Increased antibody-dependent cellular cytotoxicity (ADCC) by concabodies (T-C, R-C, O-C) compared to wild-type antibodies(n=3). ADCC was measured by co-incubating 1×10^4^ Raji cells with 5×10^4^ healthy donor PBMCs. **(L)** Enhanced B-cell depletion by concabodies compared to wild-type antibodies in ex vivo assays using PBMCs from patients with chronic lymphocytic leukemia (CLL). Data are presented as mean ± SD. Statistical significance was determined using Welch’s t-test for comparisons between parental controls and specific groups **(G, H)** .For comparisons between multiple conjugate groups, One-way ANOVA followed by Tukey’s *post-hoc* test was employed. **(F–J)** Significance is indicated as follows: ns, not significant; *p < 0.05; **p < 0.01; ***p < 0.001; ****p < 0.0001. #p < 0.05, ##p < 0.01, ###p < 0.001, ####p < 0.0001.

To test this hypothesis, we generated concabodies by fusing Con A to the Fc region of anti-CD20 antibodies following a strategy similar to that used for APEX2 fusion. The expression and purity of the resulting concabodies were confirmed (**Figure 3A**). Notably, unlike trastuzumab (TRA)-Con A fusion antibody, concabodies derived from obinutuzumab (OBI) and rituximab (RTX) demonstrated significantly enhanced DCD in a dose-dependent manner compared to their respective wild-type antibodies (**Figure 3B**). In consistent with enhanced DCD, LMP was also significantly enhanced in both Raji and Ramos cells (**Figures 3I, J**). Although TRA-Con A (T-C) showed some cytotoxicity at higher concentrations (21 nM T-C, 4.8 ± 1.21% cell death) and 70 nM T-C, 6.9 ± 2.9% cell death), this was minimal compared to the toxicity of Con A alone(**Figure 3C**). Consistent with these results, concabodies exhibited minimal cytotoxicity in CD20-negative cells, further supporting the role of antibody-mediated targeting in mitigating the non-specific effects of Con A. For example, OBI–Con A (O-C) showed only 6.8% ± 1.2% and 10% ± 1.5% cell death even in the high concentration at 146 nM and 488 nM, respectively. Furthermore, in a separate replicate experiment at 210 nM, OBI–Con A exhibited slightly higher cytotoxicity compared to OBI alone, however, this difference was not statistically significant (**Figure 3D**). These results indicate that the general non-specific lectin toxicity of Con A was substantially reduced in the concabody format, contributing to increased target specificity and safety. Furthermore, we compared the DCD induced by concabodies with that induced by co-treatment with Con A and anti-CD20 antibodies. Remarkably, concabody exhibited significantly greater DCD than the co-treatment conditions, suggesting a synergistic interaction between the antibody and the conjugated Con A moiety (**Figure 3E**). Together, these results demonstrate that fusing Con A with anti-CD20 antibodies enables the effective targeting of CD20-positive cells, enhances the cytotoxic potential of the antibodies, and reduces the off-target toxicity associated with Con A.

To further elucidate the role of MPZL1 in concabody-mediated cell death, we employed doxycycline(DOX)-inducible MPZL1-knockdown Raji cells. In this context, the cytotoxic effects of both RTX-Con A(R-C) and OBI-Con A(O-C) were substantially attenuated relative to those observed in the wild-type cells. Notably, the cytotoxic activity of OBI-Con A(O-C) was almost completely abrogated by MPZL1 silencing, whereas RTX-Con A(R-C) retained its partial activity. These findings suggest that the two fusion proteins may have distinct mechanisms of action (**Figure 3F**). To investigate whether the enhanced LMP and DCD induced by concabodies are mediated through Con A-driven internalization, we compared the internalization efficiency of concabodies with their corresponding wild-type anti-CD20 antibodies. As shown in **Figure 3G**, concabodies demonstrated significantly increased internalization efficiency, implying that Con A fusion facilitates cellular uptake and may potentiate downstream cytotoxic effects, such as DCD. To assess the requirement of MPZL1-mediated signaling in concabody-induced DCD, we used Raji cells expressing the MPZL1^Y241F^ mutant, a dominant-negative form that impairs downstream phosphorylation cascades (44). These cells exhibited a comparable extent of cell death to that of empty vector (EV) controls upon concabody treatment (**Figure 3H**). This finding indicated that the enhanced cytotoxicity conferred by concabodies is not solely dependent on MPZL1 phosphorylation. Taken together, these results suggest that, while MPZL1 signaling may contribute to the cellular response to Con A, it is not essential for concabody-induced cytotoxicity. Instead, the Con A-mediated enhancement of antibody internalization appears to play a central role in promoting the efficacy of concabodies (**Figure 3G**). The differential dependence on MPZL1 observed between OBI-Con A and RTX-Con A suggests that their respective mechanisms of action may be driven by distinct internalization dynamics or downstream effector pathways (**Figure 3F**).

Previous studies have demonstrated that OBI-induced DCD can enhance antibody-dependent cellular cytotoxicity (ADCC) (40). In line with these findings, the concabodies exhibited increased DCD and ADCC activity compared to their wild-type antibody counterparts (**Figure 3K**). Moreover, concabodies induced significant B-cell depletion in peripheral blood mononuclear cells (PBMCs) derived from patients with chronic lymphocytic leukemia (CLL), with the OBI-Con A fusion showing the most pronounced effect (**Figure 3L**). Although concabodies enhanced both DCD and ADCC *in vitro*, their *ex vivo* efficacy in depleting B cells from CLL patient-derived PBMCs was particularly prominent for the OBI-Con A construct. These results highlight the therapeutic potential of concabodies as a novel strategy for augmenting antibody-mediated cytotoxicity by harnessing the synergistic effects of Con A and antigen-specific antibody targeting.

## Discussion

B-cell non-Hodgkin lymphoma (BNHL) comprises a heterogeneous group of B-cell malignancies. Obinutuzumab (OBI), a second-generation anti-CD20 monoclonal antibody, has demonstrated superior therapeutic efficacy compared to rituximab (RTX) largely because of its enhanced capacity to induce direct cell death (DCD) (11, 13). Although the phenotypic features of OBI-induced cell death, such as homotypic adhesion and lysosomal dysfunction, have been described, the underlying molecular mechanisms at the protein level remain poorly defined. In this study, we aimed to bridge this knowledge gap by identifying proteins interacting with CD20 upon OBI binding using a proximity labeling strategy, and translating these findings into a novel therapeutic approach targeting lysosome-mediated cell death in B-cell malignancies.

To better understand the basis of these differences, we analyzed the subcellular localization of proteins captured by each anti-CD20 antibody using APEX2 labeling. Our previous study demonstrated that OBI binds non-raft CD20 and induces lysosomal cell death through the suppression of STIM1, whereas RTX primarily targets raft-associated CD20 linked to complement activation (45). Consistently, APEX2-based profiling revealed that RTX enriched plasma membrane proteins, whereas OBI captured more cytoplasmic and endosomal proteins (**Figure 1I**), reflecting distinct internalization dynamics and possibly contributing to their differential cytotoxic mechanisms.

MPZL1 is an oncogenic transmembrane protein with a glycosylated extracellular domain that promotes cancer cell proliferation, migration, and invasion (21, 23, 24). Notably, MPZL1 also interacts with concanavalin A (Con A), a lectin with high affinity for glycoproteins (27). However, the role of MPZL1 in regulating cell death has not yet been fully elucidated. Our findings indicate that Con A-induced cell death, similar to OBI, represents a form of lysosomal cell death that is at least partially dependent on MPZL1. These results suggest that MPZL1 may serve as a potential therapeutic target for enhancing the efficacy of antibody-based therapies through lysosome-mediated cell death. To exploit this mechanism, we developed a Con A-antibody fusion protein, termed concabody by linking Con A to the Fc region of anti-CD20 antibodies—a strategy similar to that used in APEX2 development (**Figure 3A**). Given that Con A binds broadly to cell-surface glycoconjugates, non-specific cytotoxicity has historically limited its clinical utility (46, 47). Remarkably, concabodies demonstrated synergistic effects that surpassed those of co-administered Con A and anti-CD20 antibodies while exhibiting significantly reduced toxicity (**Figures 3B, C**). These results clearly indicate that fusing Con A with CD20-targeting antibodies significantly reduces its inherent lectin-associated toxicity. For instance, at 70nM, the fusion protein induced only approximately 7% cell death, compared to approximately 50% with Con A alone (**Figure 3C**), suggesting that antibody-mediated targeting effectively shields non-target cells from Con A exposure.

Furthermore, we demonstrated that the concabody-induced cytotoxicity was dependent on MPZL1 expression (**Figure 3F**). Using both Raji and Ramos cell lines (**Figures 3I, J**) as well as CLL patient-derived PBMCs (**Figure 3L**), we established that concabodies induce significant cell death and B cell depletion in two B-cell lymphoma cell lines and primary CLL PBMCs. While both RTX-Con A and OBI-Con A enhanced DCD in *in vitro* cell lines, only OBI-Con A was effective in depleting B cells *ex vivo* in CLL-derived PBMCs (**Figure 3L**). This discrepancy may be attributed to differences in cell line susceptibility and MPZL1 expression levels. Although a comprehensive expression profiling across a broader panel of cell lines has not yet been conducted, we speculate that the intrinsic mechanistic differences between RTX and OBI—specifically regarding their distinct epitope binding modes and Type I/II-induced cell death mechanisms—may lead to differential responses depending on varying receptor densities. These fundamental pharmacological variations likely dictate how each antibody interacts with its target under different expression levels, and we plan to investigate these dynamics more systematically in our future studies. Other contributing factors, such as cell-type-specific signaling contexts or experimental conditions, may also influence B-cell depletion efficacy.

Notwithstanding these variations, our data collectively point toward a shared underlying mechanism: the targeted engagement of MPZL1 significantly promotes the intracellular delivery of the therapeutic agent. Consequently, these findings suggest that concabody-induced internalization, potentially facilitated by MPZL1, represents a novel mechanism that enhances antibody cytotoxicity by bypassing conventional resistance and amplifying direct cell death. Although the consistent efficacy observed across three independent CLL patient samples (n=3) provides a strong proof-of-concept for the clinical potential of concabodies, we acknowledge that the limited sample size is a constraint of the current study. Given the inherent heterogeneity of CLL, future studies involving a larger patient cohort will be essential to further validate these findings and to more comprehensively evaluate the therapeutic impact across a broader spectrum of disease states and patient profiles.

In addition to DCD, concabodies also exhibited increased ADCC activity compared to wild-type antibodies (**Figure 3K**). In a previous study, we demonstrated that enhanced DCD by anti-CD20/TNF-α^mut^ bispecific antibodies augments ADCC (40). Accordingly, our data suggests that Con A fusion can potentiate both LMP- and DCD-mediated killing, even in antibodies that do not inherently induce DCD, while simultaneously boosting ADCC activity. This represents a promising strategy to overcome the limitations of conventional anti-CD20 antibodies and to generate more potent therapeutic agents. Nevertheless, we acknowledge several limitations in the current study that warrant further investigation. First, the ADCC activity was evaluated using peripheral blood mononuclear cells (PBMCs) at a fixed Effector-to-Target (E:T) ratio (**Figure 3K**). While these results clearly indicate the enhanced potency of concabodies, further comprehensive validation across a broader range of E:T ratios and the use of purified primary NK cells will be essential to precisely characterize the contribution of specific effector cell populations and to further optimize the therapeutic window. Second, while our findings demonstrate consistent cytotoxicity in Burkitt lymphoma models (Raji and Ramos) and primary CLL patient samples, validating these results across a broader range of lymphoma subtypes, such as diffuse large B-cell lymphoma (DLBCL) and other non-Hodgkin lymphoma variants, remains a crucial future direction. Furthermore, as the efficacy of our platform was primarily evaluated using anti-CD20 antibodies, expanding this engineering strategy to include diverse antibody formats or alternative protein scaffolds will be essential to broaden its therapeutic applicability. Finally, despite the translational possibilities demonstrated by the ex vivo data from patient-derived PBMCs, the absence of *in vivo* validation limits the full assessment of therapeutic efficacy and potential toxicity in complex physiological contexts. Future studies should aim to evaluate concabody performance in relevant *in vivo* lymphoma models to further support its clinical utility and safety profile. Addressing these areas will be the focus of our subsequent research to more comprehensively evaluate the therapeutic breadth and clinical versatility of concabodies.

Our results not only clarify the role of MPZL1 in antibody-induced DCD but also propose a novel therapeutic strategy for enhancing anti-CD20 efficacy. However, several questions regarding the clinical translation of this platform remain. As noted earlier, the inherent cytotoxicity of Con A, stemming from its nonspecific glycoprotein binding, poses a significant challenge. However, recent studies suggest that targeting cancer cells with high mannose expression or utilizing Con A as a scaffold for drug delivery—such as epirubicin conjugation, which demonstrated enhanced cytotoxicity and minimal systemic toxicity in preclinical bladder cancer models both *in vitro* and *in vivo*—can significantly improve targeting selectivity and the therapeutic index (48). Based on these previous findings and our current results, Con A based concabodies could serve as a versatile platform for targeted drug delivery, capable of inducing potent cell death through internalization-based mechanisms including antibody-drug conjugates (ADC). Although our data implicate MPZL1 in promoting Con A internalization and cell death, the precise mechanism and underlying signaling pathways remain to be defined. Identification of additional regulators of Con A-induced cell death could reveal novel therapeutic targets. Ultimately, addressing these mechanistic details will refine our understanding of antibody-induced cell death and advance the clinical potential of lectin-antibody fusion proteins, such as concabodies. By elucidating the role of MPZL1 in OBI-mediated cell death through antibody-APEX2 proximity labeling and demonstrating the therapeutic potential of concabodies, this study introduced a novel strategy to enhance the efficacy and specificity of antibody-based therapies. These findings lay a strong foundation for the development of next-generation antibody–protein conjugates with improved targeting and reduced off-target toxicity.

## Data Availability

The datasets presented in this study can be found in online repositories. The names of the repository/repositories and accession number(s) can be found in the article/**Supplementary Material**.
