# Supplementary material for: A novel anti-CD20, concabody, enhances immunotherapy efficacy by targeting MPZL1 and augmenting antibody-induced cell death
Source: Front Oncol. 2026 Apr 15;16:1748576. doi: 10.3389/fonc.2026.1748576 (PMC13124566; doi:10.3389/fonc.2026.1748576)
Supplement: Supplementary file 1 [file Table1.pdf]

Table S1. List of proteins identified by HPLC-MS/MS through APEX2-mediated proximity labeling. The fold-change (FC) values represent the ratio of normalized protein intensities. Ratios were calculated by dividing the normalized intensity of the Obinutuzumab-APEX2 (O-A) group by that of the Rituximab-APEX2 (R-A) group. An FC value > 1 indicates a higher relative abundance in the O-A group.

Rituximab-APEX2						Obinutuzumab-APEX2					
**FC**	**Protein**	**Subloc**	**FC**	**Protein**	**Subloc**	**FC**	**Protein**	**Subloc**	**FC**	**Protein**	**Subloc**
Rit	FCRL3	PM	0.62	SLC39A10	PM	Obi	IGHV1-69	PM	1.54	TSPAN14	PM
Rit	ATP11C	PM	0.61	PLXNA1	PM	Obi	HNRNPH1	Cyt	1.51	SH2D1A	PM
Rit	MFGE8	PM	0.61	PTPRC	PM	Obi	CORO1A	Endo	1.5	CD40	PM
0.94	ALCAM	PM	0.61	CD46	PM	Obi	ACLY	Cyt	1.47	ST14	PM
0.91	CD22	PM	0.6	CD19	PM	Obi	RAB11A	Endo	1.45	CD82	PM
0.88	SLC3A2	PM	0.59	PLXNB2	PM	7.66	LDHB	Cyt	1.44	F11R	PM
0.84	ITGA4	PM	0.57	MET	PM	6.21	RPS25	Cyt	1.36	HSP90AA1	Cyt
0.84	ATP1A1	PM	0.51	CD83	PM	4.8	TRIM21	Lys	1.34	UBC	Cyt
0.78	ITGB1	PM	0.45	SLC39A6	PM	4.43	BSG	PM	1.26	B2M	Cyt
0.78	ADGRE5	Cyt	0.37	PTPRJ	PM	2.88	HLA-E	PM	1.26	HLA-DQA1	Lys
0.76	ITGB2	PM	0.36	NOTCH2	PM	2.5	CCT7	Cyt	1.21	HLA-C	Endo
0.75	ICAM1	PM	0.36	NEO1	PM	2.22	CCT8	Cyt	1.16	CD37	PM
0.74	ADAM17	Cyt	0.34	ATP1B3	PM	2.13	PTBP1	Cyt	1.15	CD38	PM
0.73	CR2	PM	0.32	DAG1	PM	2.08	SLAMF6	PM	1.15	HLA-DRA	Lys
0.72	NRP2	PM	0.2	CANX	Cyt	2.08	RPS18	Cyt	1.12	HLA-DRB3	Lys
0.72	ADAM10	PM	0.16	TMEM30A	PM	1.86	CCT2	Cyt	1.12	HLA-A	PM
0.68	TFRC	Lys	0.14	IL21R	PM	1.77	MILR1	PM	1.07	FCGR2B	PM
0.67	IGSF8	PM	0.09	LNPEP	Lys	1.75	MPZL1	PM	1.07	SLC2A5	PM
0.66	CD72	PM	0.06	PODXL	PM	1.62	YWHAZ	Cyt	1.06	SEMA4A	PM
0.64	ECE1	PM	0.02	HLA-DQA2	Lys	1.6	VCP	Cyt	1.05		

Abbreviations: FC, fold-change; **Subloc**, subcellular localization; **PM**, plasma membrane; **Cyt**, cytosol; **Lys**, lysosome; **Endo**, endosome; **RIT**, proteins identified only in the Rituximab-APEX2 group; **Obi**, proteins identified only in the Obinutuzumab-APEX2 group.
