# Supplementary material for: A novel anti-CD20, concabody, enhances immunotherapy efficacy by targeting MPZL1 and augmenting antibody-induced cell death
Source: Front Oncol. 2026 Apr 15;16:1748576. doi: 10.3389/fonc.2026.1748576 (PMC13124566; doi:10.3389/fonc.2026.1748576)
Supplement: Supplementary file 2 [file Image1.pdf]

Supplementary Figures

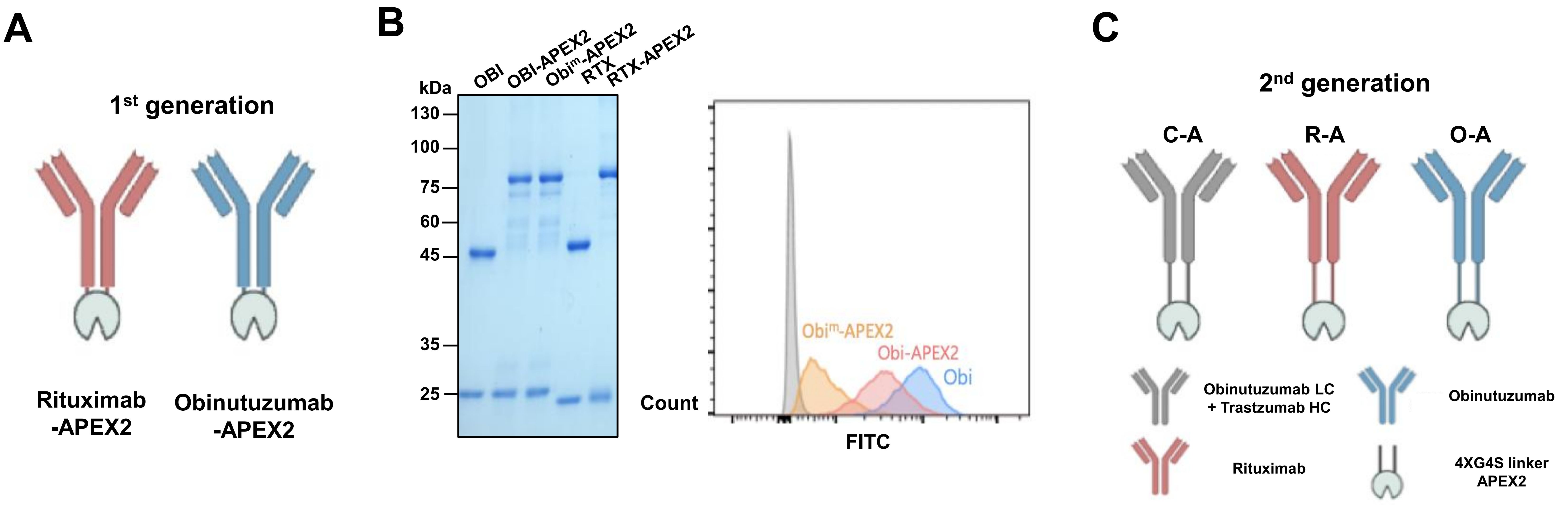

Figure S1. Optimization of APEX2-fused anti-CD20 antibodies for proximity labeling.(A) Schematic representation of the first-generation APEX2 design, where APEX2 (~27kDa) was directly fused to the Fc region of rituximab(RTX) or obinutuzumab (OBI) antibodies. Negative control antibodies (Obi^{m}-APEX2) were generated by introducing an N97A mutation in the OBI light chain (LC). (B) Validation of purified antibodies by SDS-PAGE under reducing conditions and coomassie blue staining. CD20 binding was confirmed via flow cytometry, comparing OBI, OBI-APEX2, and the negative control Obi^{m}-APEX2. (C) Design of second-generation APEX2-fused antibodies incorporating a 4×G4S linker (~2KDa), using RTX or OBI light chains and Trastuzumab heavy chains.

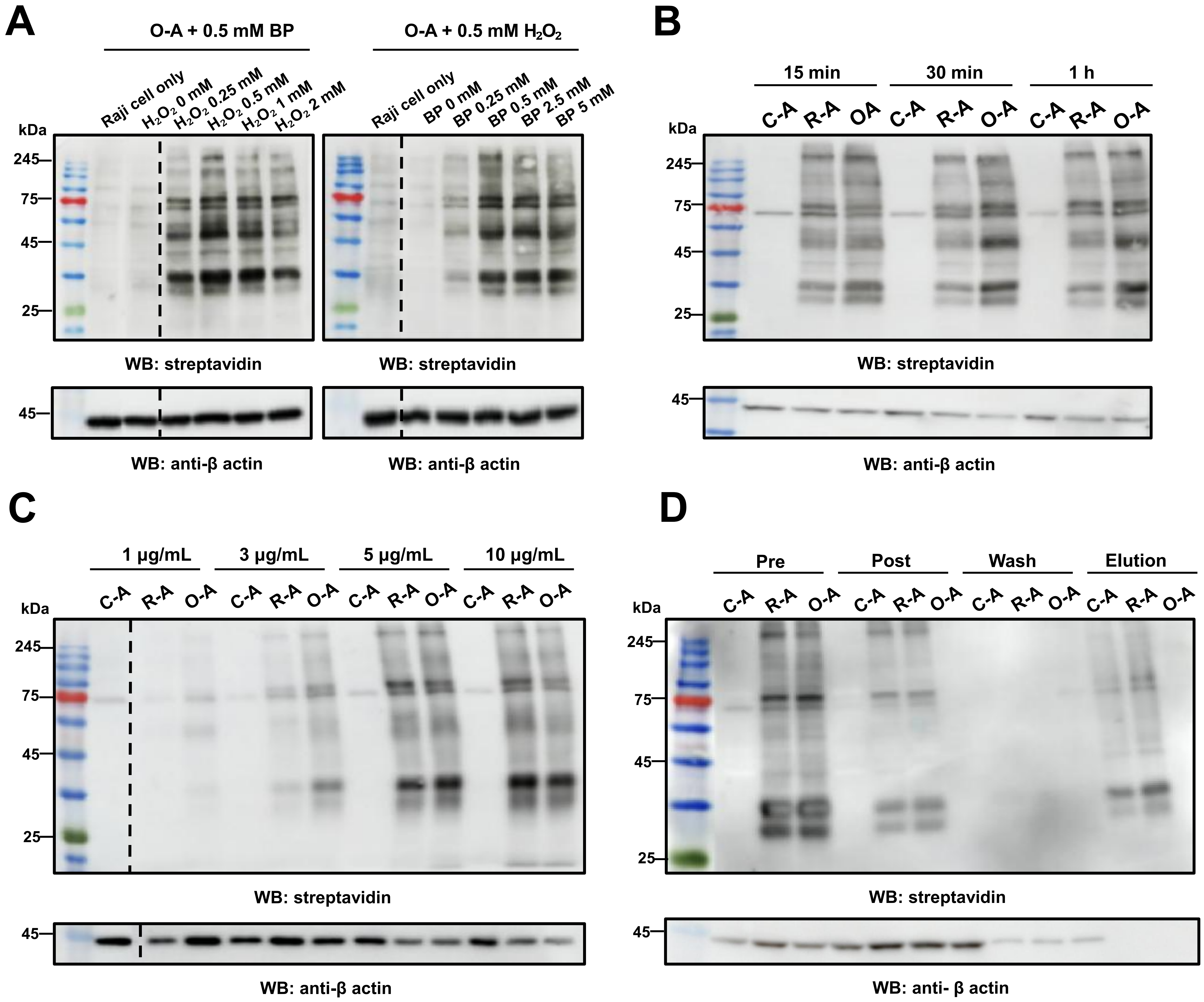
A
O-A + 0.5 mM BP
O-A + 0.5 mM H_2O_2
Raji cell only
H_2O_2 0 mM
H_2O_2 0.25 mM
H_2O_2 0.5 mM
H_2O_2 1 mM
H_2O_2 2 mM
Raji cell only
BP 0 mM
BP 0.25 mM
BP 0.5 mM
BP 2.5 mM
BP 5 mM
kDa
245
75
45
25
WB: streptavidin
WB: streptavidin
45
WB: anti-β actin
WB: anti-β actin
B
15 min
30 min
1 h
C-A R-A OA C-A R-A O-A C-A R-A O-A
kDa
245
75
45
25
WB: streptavidin
45
WB: anti-β actin
C
1 μg/mL
3 μg/mL
5 μg/mL
10 μg/mL
C-A R-A O-A C-A R-A O-A C-A R-A O-A C-A R-A O-A
kDa
245
75
45
25
WB: streptavidin
45
WB: anti-β actin
D
Pre
Post
Wash
Elution
C-A R-A O-A C-A R-A O-A C-A R-A O-A C-A R-A O-A
kDa
245
75
45
25
WB: streptavidin
45
WB: anti- β actin

Figure S2. Optimization of biotinylation conditions for proximity labeling. (A–C) Evaluation of biotinylation efficiency and patterns via western blotting under various conditions. (A) Titration of biotin phenol (BP) and H_2O_2 concentrations, where OBI-APEX2(O-A) antibodies were incubated with different concentrations of BP (0, 0.25, 0.5, 1, 2.5 mM) or H_2O_2 (0, 0.25, 0.5, 1, 2 mM) for 5 min, followed by the addition of 0.5 mM BP and H_2O_2 for 1 min. (B) Biotinylation efficiency assessed over different incubation times (15 min, 30 min, 1 h). (C) Concentration-dependent effects of the antibody (1 µg/mL, 3 µg/mL, 5 µg/mL, 10 µg/mL) on biotinylation efficiency. (D) Western blot analysis of proximity-labeled proteins using 5×10^6 Raji cells incubated with 5 µg/mL of each antibody and 0.5 mM BP and H_2O_2. Samples were analyzed across pre-incubation, post-incubation, washing, and elution steps. Representative blots are shown (n=2). Vertical dashed lines indicate where non-adjacent lanes from the same blot have been spliced together.

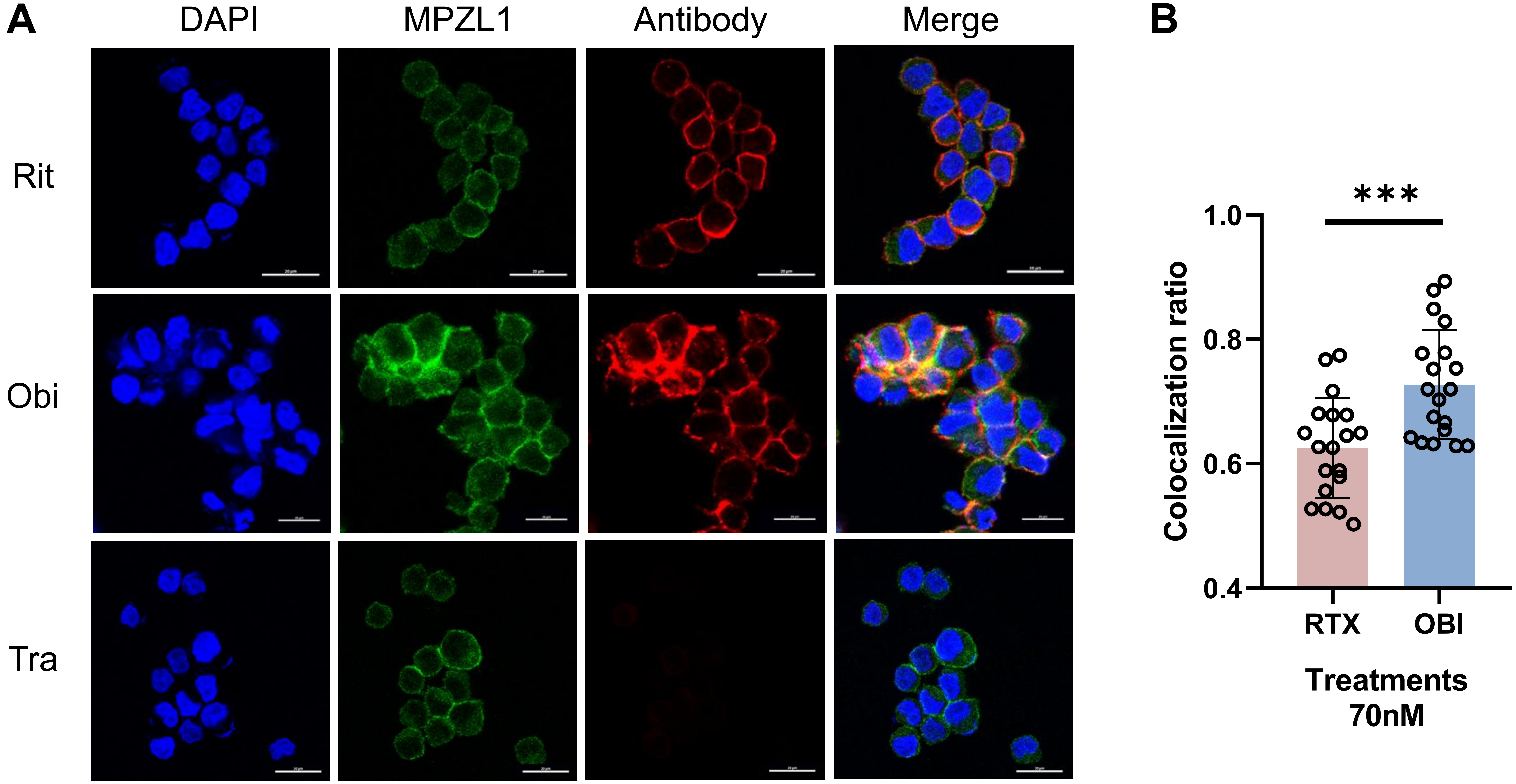

Figure S3. Colocalization of anti-CD20 antibodies with MPZL1 at the plasma membrane. (A and B) Representative confocal microscopy images of Raji cells treated with the indicated anti-CD20 antibodies (70 nM). Nuclei were counterstained with DAPI (blue); MPZL1 and anti-CD20 antibody signals are shown in green and red, respectively. Merged images (yellow) indicate colocalization of MPZL1 and the antibodies. The degree of colocalization (B) was quantified using the thresholded Manders' overlap coefficient (tM2) via the Coloc 2 plugin in Fiji/ImageJ. To account for non-specific background, the baseline colocalization level was defined using a control antibody (Trastuzumab), and quantitative analysis was restricted to cells exceeding this threshold. Each dot represents an individual cell pooled from two independent biological replicates (n=19). Data are presented as mean ± SD. ***P < 0.001. Scale bars, 20μm.
